# Complete Genome Sequence of *Photobacterium damselae* subsp. *piscicida* Strain OT-51443 Isolated from Yellowtail (*Seriola quinqueradiata*) in Japan

**DOI:** 10.1128/genomeA.00404-17

**Published:** 2017-05-25

**Authors:** Takashi Aoki, Yuki Teru, Natsuki Morimoto, Tomoya Kono, Masahiro Sakai, Tomokazu Takano, John P. Hawke, Yutaka Fukuda, Haruko Takeyama, Jun-ichi Hikima

**Affiliations:** a Integrated Institute for Regulatory Science, Research Organization for Nao and Life Innovation, Waseda University, Tokyo, Japan; b Department of Biochemistry and Applied Biosciences, Faculty of Agriculture, University of Miyazaki, Miyazaki, Japan; c National Research Institute of Aquaculture, Japan Fisheries Research and Education Agency, Minami-Ise, Mie, Japan; d Department of Pathobiological Sciences, School of Veterinary Medicine, Louisiana State University, Baton Rouge, Louisiana, USA; e Fisheries Research Division, Oita Prefectural Agriculture, Forestry and Fisheries Research Center, Saiki, Oita, Japan; f Department of Life Science and Medical Bioscience, Waseda University, Tokyo, Japan

## Abstract

Pseudotuberculosis caused by infection of *Photobacterium damselae* subsp. *piscicida* has caused serious economic damages to aquaculture farms worldwide. Here, the whole-genome sequence of *P. damselae* subsp. *piscicida* strain OT-51443, isolated in Japan, was determined and suggests that this genome consists of two chromosomes and five plasmids.

## GENOME ANNOUNCEMENT

*Photobacterium damselae* subsp. *piscicida* is a Gram-negative bacterium responsible for acute fish pseudotuberculosis, a very serious bacterial septicemia. This infection is one of the most threatening bacterial diseases in mariculture worldwide due to its wide host range, massive mortality of infected fishes, ubiquitous geographical distribution, and widespread antibiotic resistance (1). Pseudotuberculosis was first observed in wild white perch (*Morone americanus*) and striped bass (*Morone saxatilis*) in 1963 at Chesapeake Bay, USA (2). It has since been observed in cultured yellowtail (*Seriola quinqueradiata*) in Japan and was confirmed to be caused by *P. damselae* subsp. *piscicida* (3–5). It was later reported in cultured gilthead sea bream (*Sparus aurata*) in Spain (6) and in sea bream and sea bass in Europe (7). To understand the dispersion mechanisms of drug-resistant genes, virulence factors, and pathogenesis of *P. damselae* subsp. *piscicida*, it is very important to have genetic information of the whole genome. However, only draft genome sequences of *P. damselae* subsp. *piscicida* (one Japanese strain, P97-008 [8]; two Spanish strains, DI21 [GenBank accession no. AKYG01000000] and L091106-03H [accession no. MCFX02000000]) have so far been disclosed. In this study, the whole-genome sequence of *P. damselae* subsp. *piscicida* strain OT-51443 was completely determined.

*P. damselae* subsp. *piscicida* strain OT-51443 was isolated from yellowtail in Oita, Japan (9). Strain OT-51443 was cultured in heart infusion broth at 25°C for 20 h. Bacterial cells were collected by centrifugation at 8,000 × *g* for 10 min. Genomic DNA was extracted from the bacterial pellets according to a previously described protocol (10). The nucleotide sequences of *P. damselae* subsp. *piscicida* were determined using the PacBio RSII sequencing platform (Pacific Biosciences) analyzed at the Macrogen Japan Co. The sequence data were assembled using HGAP2, Falcon, and PBcR. Genome annotation was conducted using the Prokka Genome Annotation BaseSpase App (11).

The number of reads and total number of bases on OT-51443 strain were 235,372 and approx. 1,562 Mb, respectively. The genome of OT-51443 consists of two circular chromosomes, named Ch1 (3,132,149 bp, 41.6% G+C content) and Ch2 (1,010,592 bp, 39.3% G+C), and five plasmids with a total size of 4,547,251 bp. Ch1 contains 3,164 coding sequences (CDSs), 129 tRNAs, and 44 rRNAs, and Ch2 contains 1,429 CDSs and three tRNAs. Among the five plasmids, two large plasmids are 145,279 bp and 138,806 bp long (190 CDSs and 178 CDSs, respectively), and the remaining three plasmids are 55,613 bp, 39,759 bp, and 25,053 bp long (59 CDSs, 52 CDSs, and 35 CDSs, respectively). This is the first complete genome sequence reported for any *P. damselae* subsp. *piscicida* strain.

### Accession number(s).

The complete genome sequence of *P. damselae* subsp. *piscicida* OT-51443 was deposited in the DDBJ/GenBank under accession numbers BDMQ01000001 to BDMQ01000007 (total of 7 entries).
